# Sodium Chloride in Food

**DOI:** 10.3390/foods14152741

**Published:** 2025-08-06

**Authors:** Sylwia Chudy, Agnieszka Makowska, Ryszard Kowalski

**Affiliations:** 1 Department of Dairy and Process Engineering, Faculty of Food Science and Nutrition, Poznań University of Life Sciences, 60-637 Poznań, Poland; 2 Department of Food Technology of Plant Origin, Faculty of Food Science and Nutrition, Poznań University of Life Sciences, 60-637 Poznań, Poland; agnieszka.makowska@up.poznan.pl; 3 Department of Meat Technology, Faculty of Food Science and Nutrition, Poznań University of Life Sciences, 60-637 Poznań, Poland; ryszard.kowalski@up.poznan.pl

**Keywords:** salt, sodium chloride, NaCl

## Abstract

Sodium chloride is a chemical compound that has been encountered by people for thousands of years, and plays a significant role in their lives. The aim of this article is to provide a comprehensive review of table salt from the perspective of health, food technology, and cultural heritage. The article discusses salt extraction and production, its composition and consumption, and its effects on the human body. The authors draw attention to new trends, such as the use of micronized salt, microencapsulated salt, and salt with colors and shapes that differ from those of typical table salt. Scientific studies on the presence of undesirable substances and the use of salt additives were reviewed. The role of salt in dairy, meat, and bakery technology was illustrated. Gaps in research on salt were highlighted. In the last part, all types of salt with geographical indications are shown. The paper suggests that producers with a long tradition in the salt sector should apply for the European geographical indications to enhance their national and cultural heritage and promote their region. The review highlights the need for further research on all aspects discussed.

## 1. Introduction

Over centuries, salt has transformed from a luxury good to a cheap, common product. In ancient times, it was a key commodity that often determined survival. Salt was used by humans to preserve food and as an additive to dishes. The expansion of salt utilization occurred only in the past two hundred years. The use of salt as a raw material in various industries has changed the structure of its use [1]. The chemical industry primarily utilizes salt for synthesizing chlorine, sodium hydroxide, sodium carbonate, hydrochloric acid, and various chlorides, along with other chemical substances. On a global scale, the consumption of salt for this purpose exceeds 60% of its production. Various industries, including refining, textiles, tanning, dyeing, paper, pharmaceuticals, cosmetics, metallurgy, and agriculture, utilize salt in smaller doses [2]. In countries with negative temperatures, salt is used to remove snow and ice from road surfaces [3]. In the United States, approximately 41% of total salt consumption is used for road de-icing, while only 4% is used for food processing [4].

The World Health Organization (WHO) recommends that adults consume less than 5 g of salt (less than 2 g of sodium) per day [5]. The 2013 WHO plan assumed a 30% reduction in salt consumption by the population over 12 years (by 2025). By the middle of that period, in 2019, no country had achieved the target level. Actions to reduce salt consumption are multifaceted. They include collaboration with the food industry, consumer education, changes to labeling systems, and salt taxes [6]. Data collected by Jeanke et al. [7] indicate that salt content can be reduced in two food products without significantly impacting consumer acceptability. These are bread and processed meat, where reductions of approximately 40% and 60%, respectively, are possible. No such clear results were obtained for other products. The study by Bobowski et al. [8] demonstrates that individuals with high hedonistic sensitivity struggle to adapt to the taste of products with reduced salt content.

Research conducted by Denver et al. [9] shows that salt is not as important to Danish consumers as other dietary recommendations, such as consuming vegetables and whole grains, and limiting sugar and fat intake. Salt content is a secondary factor when choosing products.

Salt has also found other uses as a medicinal agent. The most commonly used salt treatments include baths (with a recommended NaCl concentration of 1.5–6.0%), inhalation therapies (with a concentration of 0.3 to 3.0%), and speleotherapy [10]. Crenotherapy (drinking mineral waters) is seen less frequently compared to other methods. Natural brines are classified as therapeutic mineral waters, distinguished by their chemical and microbiological purity, as well as the inherent variability in their physical and chemical characteristics. These are underground waters with a total mineralization of at least 35 g/L, the main components of which are chloride and sodium ions. Water with a NaCl concentration of >1.5% is called brine, while below this value, salt water [11]. A salt concentration in water of 1.0–1.5% stimulates intestinal peristalsis and the secretion of digestive juices. The effectiveness of brines in therapy relies on their enhanced composition of bioelements (biophilic elements), particularly calcium, iodine, bromine, magnesium, boron, potassium, and lithium [12].

The aim of this article was to describe salt from various perspectives, with a particular focus on its effects in food. Economic, chemical, and nutritional aspects were discussed. A separate chapter was devoted to bread, meat, and dairy products, as they constitute the main sources of salt in the human diet in many countries [13]. Moreover, new risks associated with salt consumption and new trends in science and practice related to salt are described. The research gaps were identified, and the potential benefits of further research in this area were highlighted.

## 2. Salt Mining and Production

One of the oldest rock salt mines in the world was discovered in Azerbaijan (in Duzdagi). Scientists have proven that salt has been mined there since 5000 BC [14]. One of the oldest active salt mines is located in Poland. It is “Wieliczka”. Its exploitation on an industrial scale (using mining methods) lasted from the 1380s to 1996. Currently, salt is obtained in this plant exclusively from the evaporation of brine from mine waters [15].

Worldwide salt production exceeds 270 million tons per year (Table 1). Salt is obtained in the following ways:

- From seawater, salt lakes, and their sediments (solar salt/pan salt);
- From brine (tunnels are drilled, water is added, the brine is pumped out, and then dried)—brine salt;
- From underground mines (rock salt).

The leading salt-producing countries are China, the United States, India, and Germany. Most of the salt is obtained from seawater, by evaporation. About one-sixth of its global production comes from underground mines, and the main producers are the United States, Canada, Germany, Pakistan, Belarus, and Italy [16].

In 1900 and 1905, India was the leader in salt production, generating 1.0 million tons and 1.2 million tons of salt, respectively [17].

A characteristic feature of mines is their longevity. The age of mines often exceeds 100 years. The Heilbronn mine (Germany) is one of the oldest active salt mines in Europe. Its construction began in 1881, and mining began on 4 December 1885. In 1984, the mine was merged with the neighboring Kochendorf mine. Since then, the mine has been the main German producer of rock salt and evaporated salt [18].

One of the recently opened mines in Europe is the Bądzów salt mine “KGHM Polska Miedź SA” (in Poland). The mine has been granted a concession to extract rock salt for 50 years, i.e., until 2063. Urbański and Gawlik described the characteristics of this mine and the modern equipment used to extract rock salt deposits [19].

**Figure 1:**
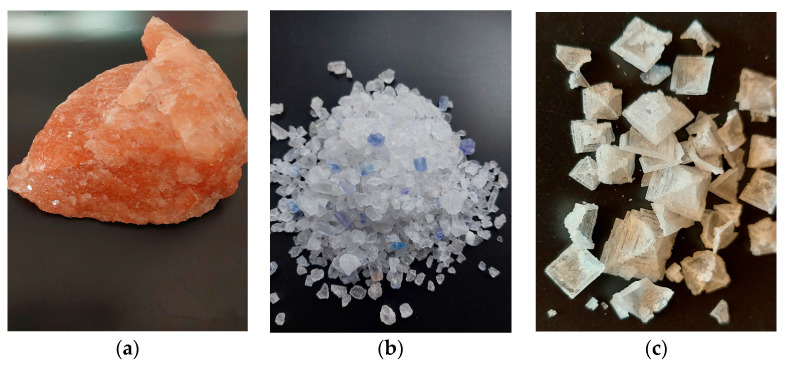
(**a**–**c**) Different colors and shapes of salt (S. Chudy).

Rock salt is transparent. There are, however, exceptions to this general rule. Pink salt is mined in Pakistan (commonly known as “Himalayan salt”) and also in Poland, in Kłodawa. The pink hue is caused by a small amount of iron oxide impurities present in the salt (Figure 1a). Another case is the blue-tinted salt from Iran (“Persian salt”) and from Poland, shown in Figure 1b (both pink and blue salts occur in the Kłodawa mine, also).

Disturbances in the crystal lattice give the impression of a color ranging from blue to navy blue. This was first described by the Polish researcher Kreutz. During the studies of salts from Stassfurt, Hallstadt, Hallein, and various samples from Kałuża, he found that blue salt does not contain any additives and its color is due to an optical effect [20]. Nassau identified different categories of imperfections related to color centers in ionic crystals [21]. A considerable number of researchers are still engaged in efforts to provide a more comprehensive analysis of electron anomalies occurring in salt [22,23]. The color and shape of salt crystals also depend on the conditions of purification and drying. Sodium chloride crystals, which grow very rapidly from a highly supersaturated solution, form a pyramidal form—a hopper crystal (Figure 1c). Such pyramids consist of a series of connected miniature cubic crystals [24].

A relatively new trend in food technology is the use of micronized salt (powdered salt, ground salt). The micronization process involves crushing salt particles (usually mechanically). So far, this process has most often been used in the pharmacy. Depending on the device and the process parameters used, very fine (10–100 µm), superfine (1–10 µm), and ultrafine (0.1–1 µm) particles can be obtained [25]. Micronized salt is available on the market, but there is no clear definition that specifies the maximum sizes of particles in such salt. In scientific studies [26,27], to obtain micronized salt, scientists used 60-mesh sieves. The size of the sieved crystals was less than 250 µm.

Micronized salt allows for a reduction in the amount of salt in a finished product. The dose of micronized salt compared to table salt can be reduced by up to half while still achieving a comparable saltiness. Araújo et al. [26] produced a fresh sausage with 50% less salt that was not sensorially different from traditional sausage.

Therefore, micronized salt can be used to produce products with reduced salt contents, which is currently a strong trend.

Fan et al. [28] presented a proposal for a low-sodium “micronized salt composite” technology. It was developed by integrating whey protein isolate, milk fat, and micronized NaCl. The new product was additionally characterized by greater resistance to clumping.

The methods of obtaining salt influence its composition (quantity and quality of impurities).

## 3. Salt Composition

Rock salt is a sedimentary rock, and its composition depends mainly on geological factors. Comparative studies of Polish and Himalayan salts have shown that salts of both types have an almost identical proportion of insoluble matter. Polish rock salt is characterized by a purity of 98.9% (1.1% of the insoluble part), and Himalayan salt is characterized by a purity of 98.8% (1.2% of the insoluble part). Salts of both types have a comparable chemical composition. Polish rock salt is richer in elements such as calcium, chromium, manganese, cobalt, strontium, rubidium, and barium. In contrast, Himalayan salt offers higher levels of potassium, magnesium, iron, copper, zinc, arsenic, selenium, and lead. Both types of salt have an almost identical content of rare-earth elements [29].

Jaworska and Niedzielski [30] analyzed six types of salt from six different countries. A high or the highest content of Mg, K, S, Si, Fe, Al, and Cr ions was recorded in the sea salt from France, which is produced from the water of the Atlantic Ocean. The daily intake of sea salt from France can provide about 10% of the daily magnesium requirement and about 2% of the iron requirement. Dead Sea salt can provide just under 10% of the daily manganese requirement. Table salt is the main source of sodium and chloride ions. Considering the daily level of salt consumption of about 5 g, it is not a key source of other essential macro- and microelements in the human diet.

The intentional addition of certain chemicals to table salt includes potassium ferrocyanide, potassium iodide, or iodate, as well as fluoride. Salt has hygroscopic properties, and in highly humid of air (75% and above), it binds water from the air and starts to clump. To reduce this phenomenon, some manufacturers add anti-caking (anti-agglomerating) substances to salt, e.g., potassium ferrocyanide (E536) in amounts of up to 8 mg/kg. However, the scientific literature indicates the harmfulness of this additive. The study by Basu and co-workers [31] demonstrated that potassium ferrocyanide exhibits toxic effects on cells and genetic material at concentrations ranging from 0 to 10 mM, while sodium ferrocyanide is not harmful to genetic material.

Table 2 lists salt additives (with anti-caking properties) that are suitable for salt, according to Codex Alimentraius [32].

Due to the general deficiency of iodine in food—apart from in certain areas with significant seaweed intake and iodine-rich water—the iodization of salt becomes necessary for adequate human development. Currently, almost 90% of the global population uses iodized salt [33]. The EU does not have a single standard for all member states regarding salt iodization. In the 124 countries where iodization is mandatory, national standards apply. These standards specify the compounds to be used and their amounts [34]. The 2014 WHO guidelines recommend potassium iodate or potassium iodide as iodine compounds for salt fortification [35]. Polish rules specify that for every 100 g of table salt, there should be 2.3 ± 0.77 mg of iodine present. This amount is equivalent to 30 ± 10 mg of potassium iodide or 39.0 ± 13 mg of potassium iodate per kilogram of salt [36]. Potassium iodate (KIO_3_) is more stable than iodide (KI) and is more commonly used by manufacturers. Potassium iodide oxidizes to iodine, which is lost from the product through volatilization. Some countries allow the use of compounds other than those recommended by the WHO [34]. In Latvia, four compounds are permitted—sodium iodide (NaI), potassium iodide (KI), sodium iodate (NaIO_3_), and potassium iodate (KIO_3_) [37].

Potassium iodide is effective for the prophylaxis of iodine deficiency [38]. Scientists from the Medical University of Łódź (Poland) have proven that potassium iodide, used in doses generally recommended for iodine prophylaxis, increases the concentration of iodine in the thyroid gland, which can prevent its oxidative damage. Research indicates that potassium iodate lacks any direct positive impact on thyroid membrane lipids, and its effectiveness in iodine prophylaxis is not indisputable [39].

Iodine overdose has a toxic effect on the body. A concentration of KIO_3_ solution between 187 and 470 mg/kg body weight can damage eye cells [40]. The opposite conclusions resulted from the experience of Li et al. [41]. They proved (in an experiment on rats) that the body can utilize high doses of KIO_3_ ingested through the digestive tract.

An in-depth, detailed study is needed in the context of substances added to salt. Further steps may be needed to verify and amend regulations in this area.

Salt can also be fluoridated. In order to prevent tooth decay, the first attempts to add this element to salt appeared in Switzerland in 1956. After 26 years, the possibility of using it as an additive to salt was officially announced. In Switzerland, 85% of the salt used is fluoridated, and in Germany, 67%. The fluoridation of salt is the most cost-effective approach to prevent cavities in teeth [42].

In Latvia, fluoridated salt, sodium fluoride (NaF) or potassium fluoride (KF) is permitted. The fluoride ion content should be in the range of 90–295 mg/kg [37]. Also in France, fluoridated salt is authorized (at a concentration of 250 mg KF/kg) [43].

**Figure 2:**
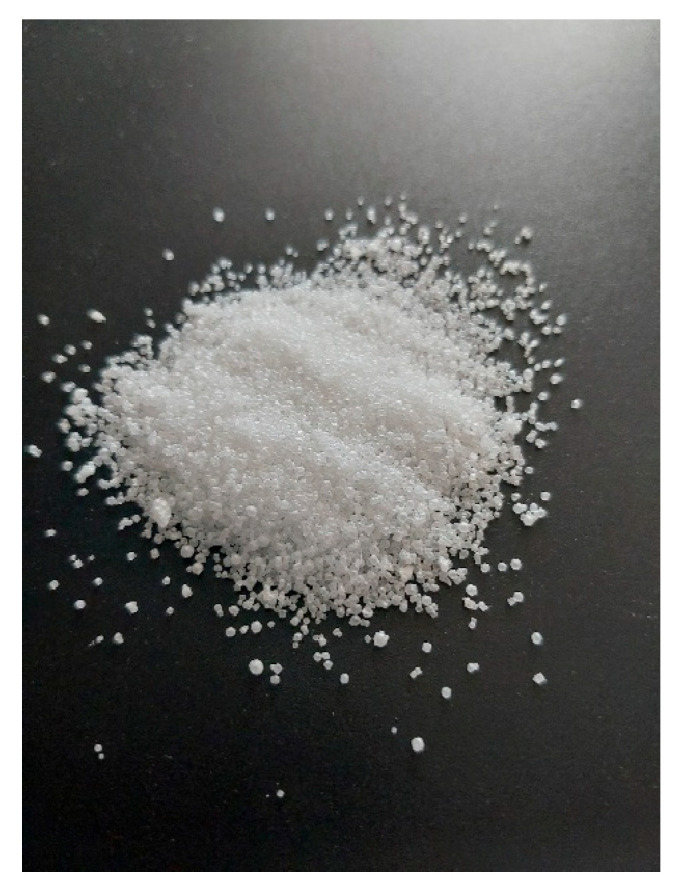
Encapsulated salt (S. Chudy).

Salt used in the food industry can be encapsulated (Figure 2). Encapsulation is a technique by which a given substance or mixture of substances (core) is coated or entrapped within another material (shell/capsule). The choice of encapsulation material is crucial and depends on factors like the release mechanism, compatibility with the core material, and cost. Various techniques can be employed to encapsulate salt, depending on the desired properties of the final product and the application. Some common methods include coating with fats or waxes. Molten fats or waxes are used to coat salt crystals, creating a barrier that melts upon heating, e.g., carnauba wax and hydrogenated vegetable oils [44].

Salt can initiate the emergence of problems related to the loss of meat juiciness. Adding salt at the initial stage of heat treatment can lead to the dehydration of the meat, which will make it dry and hard. This effect of salt is caused by its loosening effect on the tissue structure of the meat. The solution to this problem may be the use of encapsulated salt. The core of such a capsule does not come into direct contact with the meat, and the shell, made of a properly selected material, liquefies at a specific temperature. Encapsulating salt offers several key advantages, such as controlled release, the prevention of premature reactions, improved texture and appearance, and flavor enhancement, or masking. The coating on encapsulated salt can be engineered to release the salt precisely when needed. This could be triggered by specific conditions like heat, moisture, or pressure, or designed to release over a set period. Encapsulation keeps salt from interacting with other ingredients too soon. For instance, in food, it stops salt from drawing out moisture prematurely, leading to a juicier end product and preventing undesirable reactions with other components. By controlling when and where salt dissolves, encapsulation can enhance the texture of food and prevent surface discoloration. A great example is how encapsulated cooking salt prevents meat from drying out. Regular salt pulls moisture from meat, making it tough and less succulent as it penetrates the tissue [45]. The encapsulating material can enable the gradual release of salt flavor, offering a better taste experience. Sometimes, the encapsulation material itself can even add to the overall flavor profile [46].

In recent years, scientists have been adding vitamins and minerals to salt in their research experiments, such as folic acid, iron, zinc, cobalamin (vitamin B_12_), and other microelements. Many studies have aimed to develop salt fortification methods, test the stability of added compounds, and assess the sensory acceptability of salt. Such studies are carried out on supplementing microelement deficiencies by salt intake in some regions of the world (e.g., India) in the future [47,48].

Some elements, such as lead, cadmium, or mercury, are highly undesirable in food products due to their toxicity and strong mutagenic or carcinogenic effects. The maximum permissible contents of these elements in food products were established. For salt, they are Pb—1 mg/kg, Cd—0.5 mg/kg, and Hg—0.1 mg/kg [49]. Jaworska and Niedzielski [30] determined the contents of Pb ions in salt from Peru (salt from an inland water reservoir) at 1.70 mg/kg. This value exceeded the established upper limit of the presence of this metal in salt by as much as 70%.

Salts from Europe, Asia, and America were checked for lead content by Salvo et al. [50]. In all the samples tested, the lead level exceeded the standards. The lowest content of it was found in smoked salt (5.24 ± 0.28 mg/kg), and the highest was in Persian blue salt (9.24 ± 0.42 mg/kg).

In recent years, researchers have also been checking the levels of nano- (particles ˂ 1 µm) and micro- (particles ˂ 5 mm) plastics in salts [51,52]. Regardless of the type of salt origin (including rock salt), microplastics were present in it. They get into the final product during the extraction and packaging processes. Developing methods to remove microplastics from salt or methods for “clean” production and packaging seems to be one of the most important needs. There are probably no more microplastic-free foods. They are present even in fruits and vegetables [53]. Their daily intake, as reported by Lehel and Murphy [54], has a potential carcinogenic effect, and disrupts the work of the liver and endocrine glands. Microplastics may contribute to the accelerated extinction of species, a process that is currently being observed [55]. Consequently, it is necessary to inform the public about the need to limit the use of plastic packaging, especially single-use packaging, as well as to promote the segregation and processing of waste and the development of technologies for removing contaminants from food [56].

The composition of salt, especially the sodium content and any additives (unintentional and intentional), has a direct impact on its effects on human health.

## 4. Salt Consumption and Body Reaction to Intake

In the past, salt consumption was very high in some regions. As Ritz cites, ancient Roman patricians consumed about 25 g of salt per day. However, not all of this salt was consumed. Some of it was lost in the water used for cooking. In 18th-century France, daily salt intake varied from 13 to 15 g depending on the region. Scandinavian countries exhibited a much higher use of salt. In Sweden during the 16th century, individuals consumed nearly 100 g of salt each day, primarily sourced from preserved fish and cured meats [57].

The share of direct salt consumption in relation to salt production is 12% and 19% in Poland and worldwide, respectively. Global salt consumption in this context is overstated by underdeveloped countries, where salt is used practically 100% for food purposes [58]. According to data included in publications from the years 2010–2021, in many countries (*n* = 52), men consume more salt than women. Salt intake ranges from 5.39 to 18.51 g per day for men and from 4.27 to 16.14 g per day for women. It is noted that the lowest salt intake is in Northwestern European countries, while the highest is in Eastern European and Central Asian countries. Malta, Cyprus, and Estonia are the countries with the lowest average daily salt intakes in Europe—4.92 g, 5.19 g, and 5.21 g per person, respectively. The highest salt intakes are observed in Kazakhstan and Kyrgyzstan—17.24 g/person, and the Russian Federation—14.87 g/person [59].

A low level of salt intake may help people taking antihypertensive medications discontinue or reduce their intake. However, there are doubts about the beneficial effects of reducing sodium intake on overall health [60]. In patients with mild to moderate hypertension and high dietary sodium intake, a diet with no added salt decreases systolic and diastolic blood pressure [61]. In healthy people, a low-sodium diet may cause insulin resistance [62].

Salt plays many very important roles in our bodies. Sodium is responsible for maintaining the proper volume and pressure of blood and the acid–base balance, and for transporting amino acids, sugars, and vitamins in tissues. Sodium ions are also necessary for the proper functioning of nerve and muscle cells, including the heart muscle, and for activating some enzymes. Sodium participates in the absorption of ingredients in the small intestine and kidneys.

The level of sodium (and potassium) ions inside the cell is lower compared to the level in the fluid outside the cell. Due to differences in ion levels between the cell and the surrounding fluid, these elements constantly move towards the region where their ion concentration is lower. As they move across the membrane, they perform the passive transfer of fluids and substances [63]. Hurley and Johnson also showed that sodium deficiency has a broad range of effects on the psyche. Sodium deficiency has negative effects on cognitive function and may induce fatigue and depressive-like symptoms [64].

Almost all sodium ion excretion (about 90%) is managed by the kidneys. The rest exits the body through feces and the secretions of sweat glands. The thyroid gland regulates sodium metabolism. If this gland is underactive, it results in the accumulation of metal ions within cells, while the heightened activity of the gland boosts sodium excretion [63].

In the human body, sodium and chlorine occur in ionic form. Their normal levels are Na^+^ 135–145 mmol/L blood serum and Cl^−^ 96–106 mmol/L blood serum. Sodium and chlorine work together to regulate the distribution of water and the acid–base balance [65]. The right level of chlorine in the human body is responsible for maintaining the right ratio of cations to anions in body fluids, which determines the pH of the blood and the correct course of all life processes. Chlorine, similar to sodium and potassium, helps control the balance of body fluids and electrolytes, ensuring proper hydration within the cells. It is vital for the absorption of vitamin B_12_ [66]. Furthermore, chlorine triggers saliva digestive enzymes, and is involved in creating hydrochloric acid in the stomach. The production of hydrochloric acid in the stomach depends on protons and chloride, but the processes and means of controlling how it is secreted in the stomach are still not well understood [67].

Until recently, the prevailing theory was that the body regulates the constant pressure of sodium ions in the blood by increasing or decreasing the total body water. A group of researchers from Germany (Heer et al.) studying healthy people did not observe an increase in the total amount of fluid in the body, or an increase in body weight with an increase in salt intake. The researchers proved that an increase in salt intake does not cause water storage in the body, but only its displacement [68].

Rabelink and Rotmans [69] proposed a model for a new explanation of Na+ balance. According to them, excess ions may be stored in the interstitium of the skin. There, sodium ions can accumulate and be released if the sodium supply is too low.

The pathophysiology of particular diseases may result from unknown causes related to the inability to store excess sodium. In 2009, Heer et al. [70] asked questions that still have no answer, concerning how much sodium the body can store, and whether there are genetic obstacles that may lead to the inability to store osmotically inactive sodium.

It should be noted that salt consumption depends largely on its presence in products and ready-made meals.

## 5. Salt in Food

Salt is one of the most commonly used ingredients in the food sector due to its properties and low cost. It is a flavor enhancer because of its effect on various biochemical mechanisms. It reduces or increases the enzymatic activity of some enzymes responsible for the development of various organoleptic parameters [71]. Table 3 lists selected products and their average salt contents.

Salt improves the foaming properties of protein preparations, and stabilizes foams and gels (Table 4). This is exploited in confectionery (production of foams, ice cream). Inyang and Iduh [76] studied the effects of salt concentration (from 0.0% to 5.5%) on the volume and stability of sesame protein concentrate foam. The highest foam volume and stability were obtained after adding a salt solution at concentrations of 5.5% and 2.8%, respectively.

An increase in the volume of egg white protein foam and a reduction in whipping time with the increase in the amount of added salt were observed by Raikos et al. [77]. Salt causes a decrease in the surface tension of egg whites [78]. The physical properties of protein or protein–fat foams (Table 4) are very widely described (depending on temperature, pH, whipping time, and salt and sugar concentration), but the descriptions of the actions of salt in these systems are still based on hypotheses [79].

**Table 4:** The effect of NaCl on the properties of protein products [80].

Product	Properties	Acting	Concentration of NaCl
Blood cell protein powder	solubility	decries	0–0.4 M; (pH = 3)
The sunflower protein	solubility c	increase	0.1 M; (pH = 4–7)
Rice protein	solubility	increase	0.1 M; (pH = 2/4.5/9
		decrease	1.0 M; (pH = 2/4.5/9)
WPI gels	water retention	increase	0.1 M; (pH = 7.5–9.5)
Pea and lentil globulins	solubility	increase	0.1–0.5%
Blood globin powder	water holding capacity	decrease	˃0.2 M
Canned frankfurters	water and fat binding	increase	5% in product
Rice protein concentrate	foaming capacity	increase	0.1 M
		decrease	1.0 M
Myosin gel (from broiler muscle)	strength	increase	0.2–0.6 M
Non-heated myofibrils	extractability of proteins	increase	0.1–0.6 M
Whey gel	hardness	increase (maximum at)	200 mM NaCI and 10 mM CaCl_2_

In water solutions, protein molecules become surrounded by water (hydration occurs). This phenomenon is possible due to the presence of polar groups in protein amino acid chains, such as carboxyl and amino groups (which interact with water via hydrogen bonds), and the polar properties of water. A sol is formed. Adding a high amount of salt to the protein solution causes the water envelope around the protein to disappear (salting out). Water molecules begin to surround the Na and Cl ions. The solution becomes denser. The protein’s solubility decreases, and a gel forms. This is a reversible coagulation process. At low salt concentrations, protein molecules become surrounded by salt ions, and these then become surrounded by water ions (salting in). An increase in protein solubility is observed. The selection of proteins and their processing conditions (temperature, salt concentration) are of key importance in modeling food matrices, as shown by Mańko-Jurkowska and Domian [81].

The influence of salt in food processing has been selectively described and explained. Furthermore, no studies have been conducted on the behavior of salt in foams depending on the system (dispersed medium (different types of gas)/dispersing medium (different types of liquid)).

### 5.1. Salt in Dairy

In dairy technology, and especially in cheese production, sodium chloride plays an important role. There are three techniques for salting cheese, namely, adding salt to the cheese grains, rubbing the formed cheese, and soaking the cheese in brine. The amount of salt added depends on the type of cheese and its size. The salt level has a major impact on the taste of cheese, its composition, and the biochemical changes during cheese maturation. The salt level affects the rheology of cheese, as well as its textural properties and production efficiency. Salt controls microbiological and enzymatic activity, and indirectly affects the aroma of cheese. The aroma components are additionally related to lactose metabolism, changes in milk fat, and casein (formation of peptides, free amino acids, and free fatty acids).

NaCl affects calcium levels, paracasein hydration or aggregation, the water-holding capacity of the casein matrix, and serum separation. The preservative effect of the salt is related to its water activity-lowering mechanism. Some water in cheese is contained within its structure and is not accessible to microorganisms [82,83].

The main function of salt is to control the moisture in cheese. Higher salt concentrations on the cheese surface cause whey to be removed and a rind to form (which is desirable). Salt determines water availability, thus influencing the growth and activity of microorganisms during cheese ripening, pH changes, enzyme activity, and the hydration of casein. A low NaCl content (5–6%, *w*/*w*) produces a softer product, which customers often associate with an unripe, inexpensive product. Increased water availability leads to more intense microbial growth, and the hydrated protein matrix is more susceptible to enzymatic hydrolysis. Therefore, it is difficult to produce low-salt cheese comparable in texture, taste, and aroma to traditional/classic cheese [84].

Salt is also added to butter. Laikoja et al. [85] evaluated the quality of salted (2%) and unsalted sweet cream butter produced in a continuous butter machine and stored at different storage temperatures for different time periods (−20 °C/24 weeks, +5 °C/12 weeks, +20 °C/8 weeks). There were significant differences between salted and unsalted butter samples in fat quality during the whole storage period, but only at −20 °C. Salted butter had a higher peroxidative value.

El Sadek et al. [86] analyzed salted and unsalted butters stored at +5 °C. The butters were made from creams with an acidity of 0.15% and 0.25%. Unsalted butters showed a better storage quality than salted butters. Unsalted butters made from sweet cream and from acid cream were stored without undesirable tastes, respectively, 120 and 150 days. Salted butters retained their initial quality up to 105 and 120 days for those made from sweet and acid cream, respectively. Regarding the acceleration of fat oxidation, some authors (after Loftus-Hijls and Conochie, 1940) have blamed this on chlorine, which forms in a reaction of hydroperoxides and hydrogen and chloride ions—R-O-O-H + 2Cl^−^ + 2H^+^ → Cl_2_ + H_2_O + R-O-H—and which induces the further oxidation of the fat.

Fitzgerald and Buckley [87] tested cheeses with salt substitutes. They found that adding MgCl_2_, KCl, or CaCl_2_ to cheese at an equivalent level to NaCl produced extremely unacceptable cheeses, often characterized by a bitter taste, metallic flavor, and crumbly body. Ganesan et al. [88] also observed that consumer preference scores decreased with reductions in salt for cheddar and low-moisture, part-skimmed mozzarella.

### 5.2. Salt in Meat

Meat salting is a method of preserving food that has been known since prehistoric times. After the introduction and popularization of cooling and freezing, it lost its importance as a preserving procedure [89]. Currently, salting, together with the addition of nitrates (III) and (V) and other ingredients (a process called curing), is used mainly to develop the desired and lasting red color and characteristic tastiness of meat, and not to increase its durability. However, to this day, salted cattle meat is an important component of the diet of some South American countries. Currently, the term “curing” refers to the effect of a curing mixture—nitrates (III or V) and NaCl—on meat. Nitrate (III) curing mixture, commonly known as curing salt, is made up of a combination of sodium chloride and sodium nitrate (III), which is the same as NaNO_2_ or E250. Nitrate (V) curing mixture is a mixture of sodium chloride and potassium nitrate (V)/KNO_3_/E 252/saltpeter. Typically, the curing mixture’s composition includes 99.4% sodium chloride and 0.6% sodium nitrate [90]. According to the applicable EU regulations, nitrate (V) can only be used in the production of cold cuts that have not undergone heat treatment [91].

The basic phenomenon occurring during curing is the process of diffusion caused by the difference in osmotic pressure between the brine and the content of the muscle tissue cells. Diffusion is possible due to the semi-permeable nature of the protein–lipid cell membranes, which allow the penetration of small ions and retain colloidal substances. In the curing process, the main role is played by Na^+^ and Cl^−^ ions. Other inorganic substances occur in small amounts compared to sodium chloride [92]. Therefore, the phenomena occurring during curing can be presented in a very simplified way as reactions occurring between a salt solution, e.g., NaR, located in the muscle tissues, and brine, separated from each other by a membrane that is permeable to ions of Na^+^ and Cl^−^ and retains R ions, symbolizing protein anions. The rate of diffusion is not constant. It depends on the kind of meat, the ratio of connective tissue to muscle, the amount of fat, the state of post-mortem changes, and the sizes of meat elements [93]. External factors, such as temperature, concentration, and degree of salt contamination, also have a significant impact. The effect of temperature is only visible on the rate of salt penetration, but it has no significant effect on the degree of salt saturation of the product. Salting with pure sodium chloride takes less time than using table salt containing impurities of other salts, such as CaCl_2_, CaSO_4_, MgCl_2_, and MgSO_4_. Salt penetration is mostly inhibited by CaCl_2_ through its effects on meat proteins [94]. In the process of salt’s penetration into the product, three phases can be distinguished, as follows:

- In the first period, osmosis, i.e., water penetration through membranes into a solution with a higher concentration, has an advantage over salt diffusion into the meat. As a result, there is a loss of mass of the raw material and a decrease in the difference in concentrations in the laminar layer on the surface of contact between the raw material and the brine. During this period, salt penetrates mainly into the outer layers of the meat. After the concentrations in the outer layers and the surrounding solution have equalized, further salt penetration takes place slowly as diffusion occurs. During this period, at low salt concentrations (up to 7%), proteins dissolve and pass into the brine;
- In the second phase, the same processes occur, but the increased salt concentration causes the denaturation and coagulation of proteins [94];
- In the third phase, the process is established. Part of the salt is bound to proteins, and the protein complexes formed cause the osmotic pressure in the tissues to become slightly higher than that of the brine. This results in the re-penetration of some water into the meat and an increase in the mass of the cured raw materials [95].

Sodium chloride is a bacteriostatic agent. Its action consists of increasing the osmotic pressure of the environment, which entails a decrease in water activity below the level that allows the growth of most bacteria. The greatest durability is achieved by meat when the salt concentration in the final product is 9–11% and the water content is 50–55%. Then, the salt concentration in the water phase of the product is close to saturation. In the case of salted bacon, on the other hand, the salt content in the outer layers is 5–8%, and due to the low water content (below 10%), 1/3–1/2 of the salt remains in solid form.

The sensitivity of microorganisms to salt varies greatly. Some bacteria cannot develop at a concentration of 2% salt, while others, as well as yeasts and molds, can withstand high concentrations, even saturation. Depending on the sensitivity of microorganisms to sodium chloride contained in the medium, they are divided into non-halophilic (halophobic and halotolerant) and halophilic [96].

In meat products, the textural effect of salt is based on increasing the electrostatic charge of the protein and facilitating the transition to dissociated, more soluble forms. Features such as the ability to bind water, i.e., protein swelling, fat emulsification, the amount of forced and thermal leakage, and water absorption, are significantly related to the salt content in the product. In meat processing, sodium chloride influences the formation of technological benefits, such as increasing water absorption and improving gelling and emulsifying properties [97]. The reasons for inhibiting the growth of microorganisms by sodium chloride include, among others, increasing the osmotic pressure and plasmolysis of microbial cells, direct toxic effect on microbial cells, reducing the solubility of oxygen in liquid environments, and weakening the activity of intracellular proteolytic enzymes [98,99]. When sodium nitrate (III) is added in addition to salt during the meat curing process, the growth of *Clostridium botulinum* and other pathogens, such as *Salmonella* and *Staphylococcus aureus*, is inhibited [100].

Steen analyzed liver paste with liver/fat ratios of 35/35 and 20/50, and with two salt levels of 0.0% and 1.8%. The addition of salt resulted in a reduction in the fat droplet size, which was also evident in the increased stability of the emulsion and notably in the enhanced fat-binding characteristics of liver paste, creating a firmer and less easily spreadable product. It was noted that the presence of salt increases the availability of proteins that can serve as emulsifiers and aid in gel formation [101].

The water-binding capacity, i.e., protein swelling, fat emulsification, forced and thermal leakage, and water absorption, is significantly related to the salt content of the product [102]. Salting alters the physical properties of meat. The improved water binding capacity is mainly due to the presence of Cl^−^ ions. They modify proteins into dissociated, and therefore more soluble, forms. Furthermore, ionic strength and pH can cause conformational modifications to the protein structure, exposing hydrophobic residues and affecting the hydrophobicity of protein surfaces [103]. This protein behavior is exploited to create protein-stabilized oil-in-water emulsions. The hydrophobicity of proteins prevents the agglomeration of fat globules, for example, in the production of hot dog sausages.

The production of homogenized meat products begins with the pre-grinding phase (particle size reduction). Grinding is necessary to break down the muscle membranes and sarcolemma. This process releases the myofibrillar proteins to allow them to swell. After grinding, salt is added, increasing the binding capacity. The addition of salt increases the net negative charge of proteins and shifts their isoelectric point (to a lower pH). Water then penetrates between the myofibrillar proteins.

It is also theorized that salt causes the repulsion of peptide chains in myofibrillar protein, allowing water to enter this space (increasing water absorption) [104].

One method for improving the water-holding capacity of meat is to process it immediately after slaughter. The benefits of “warm meat” (non-refrigeration) processing result from the specific effect of salt on the contraction process of muscle fibers. Salt added in an amount of 2–3% to meat obtained immediately after slaughter blocks the formation of bridges between myosin and actin, and significantly slows the conversion of glycogen to lactic acid. It contributes to the increased water absorption of the meat. This effect is maintained even when the salted meat is frozen or freeze-dried [105].

### 5.3. Salt in Bakery

**Figure 3:**
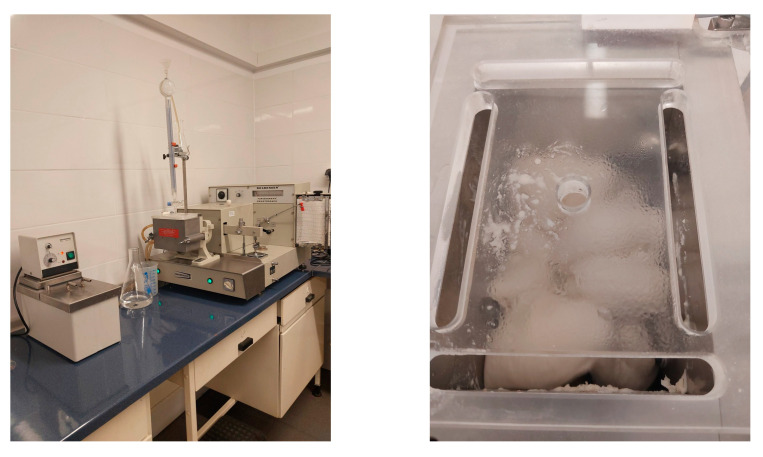
Farinograph and dough mixing during analysis (S. Chudy).

The way salt works in another branch of the food industry, baking, is also interesting. Bread is a staple food product that provides the body with about 25% of its daily salt intake [106]. The addition of salt affects not only the taste of the bread made from wheat flour, but is also a fundamental ingredient in bread making, impacting dough rheology, gluten development, and overall bread quality. Understanding its specific effects on gluten proteins, dough fermentation, and starch viscosity is crucial for optimizing baking processes. The farinograph (Figure 3) is a tool used for measuring the properties of a wheat dough.

How salt influences the rheological characteristics of wheat dough was demonstrated in the experiment. A mixture of wheat flour (300 g) along with varying amounts of salt (ranging from 0.0% to 2.5%) and water was combined in a farinograph mixer (Brabender OHG, Duisburg, Germany). Water was added to achieve a dough consistency of 500 Brabender Units (BU). The parameters assessed included water absorption (amount of water absorbed by 100 g of flour), dough development time (time to reach maximum dough consistency—500 BU), dough stability (time dough remains at maximum consistency), degree of softening (reduction in dough consistency), and the farinograph quality number (FQN—length of the horizontal segment between points 500 BU and 470 BU on the farinogram graph). The analysis was carried out according to the AACC International Method 54-21.02 [107].

In the conducted studies, it was found that the addition of salt at the amount of 1.5% did not significantly affect the water absorption of flour, nor the development time, stability time, or softening of the dough (Table 5).

However, when the amount of salt was 2.5%, the rheological properties of the dough improved. The water absorption of the flour and degree of dough softening decreased. The dough development and stability times were extended. The Farinograph Quality Number increased. The interaction of salt ions with wheat gluten proteins reduces the water-binding capacity of gluten, changing the physical state of the protein molecules. It is believed that sodium chloride masks the repulsive effect of molecular charges by interacting with the polar groups of proteins [108].

The effects of different salt concentrations on dough rheology, gluten secondary structure, zeta potential, and gliadin to glutenin ratios were investigated by Chen et al. [109]. The key finding was that salt increased macromolecular gluten aggregation, reduced free sulfhydryl (SH) content and strengthened the beta-sheet structure of gluten, which resulted in improved dough rheological properties. The presence of salt in dough affects the hydration of gluten proteins [110]. Sodium ions (Na^+^) can neutralize charges on proteins, reducing repulsive forces [111]. In this way, they promote molecular interactions and gluten aggregation [109]. Anions (Cl^−^) are generally more effective at associating with proteins and screening electrostatic repulsion at specific binding sites than cations. Anions can bind directly to the polar nitrogen atoms of positively charged amino acid residues and through anion–amide interactions with gluten proteins, influencing their structure [112]. NaCl strengthens inter-protein hydrogen bonds and hydrophobic interactions. Kuang and Xu [113] stated that the loss of repulsive forces allowed proteins to form intermolecular hydrogen bonds, resulting in aggregation. Studies suggest that high salt levels induce stronger gluten interactions via SH cross-linking [109]. The interaction of Na^+^ and Cl^−^ ions with gluten proteins is a complex process that significantly impacts the functional and rheological properties of dough. This primarily occurs through the shielding of electrostatic charges, the influence on hydration, and the strengthening of key non-covalent bonds, such as hydrogen bonds. As a result, the gluten network becomes stronger and more ordered (fibrous structure), and exhibits increased molecular rigidity, which manifests as improved rheological parameters, although gluten network formation is simultaneously delayed. These mechanisms are crucial to the quality of the final baked product [109].

Salt in bread dough enhances product flavor, strengthens the gluten network, increases the dough mixing time to increase protein–protein interactions, and acts as a preservative by decreasing water activity and prolonging shelf life. Lower salt concentrations in the dough lead to more interactions between protein and water, creating a less robust gluten network. In the absence of sufficient salt, the gluten network will not develop properly, increasing yeast activity, which ultimately results in bread that is lacking in quality regarding its texture, size, taste, and appearance [114]. Salt-free dough is not very elastic, ferments unevenly throughout the mass, sticks when processed, and is unstable. Bread without salt has a smaller volume and is harder to keep fresh. The crust is crumbly and pale, the flesh is unevenly porous, and the bread tastes bland [115].

Salt affects yeast fermentation in the dough. It typically decreases the total CO_2_ production while increasing the retention coefficient. Salt inhibits yeast activity, which is important for controlling gas production during bread-making [116].

The addition of salt can modulate the pasting properties of the wheat flour. It was observed that salt influences the amylolytic activity in flour. It was also stated that the addition of salt increases peak viscosity and falling number values [94].

**Figure 4:**
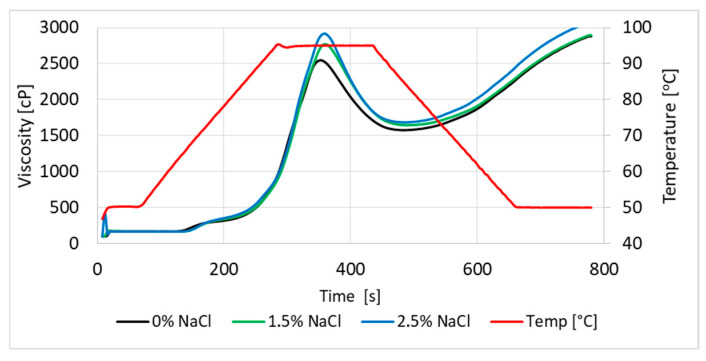
Viscosity of dough without and with salt addition.

The pasting properties of wheat flour slurries with the addition of salt (0.0–2.5% related to flour) were analyzed in the RVA Tec Master (Perten Instruments, Hägersten, Stockholm, Sweden), in accordance with the standard AACC International Method 76-21.01 [117]. Flour (3.5 g) and unsalted or salted water (25 mL) were mixed in the RVA container. The salt contents in the analyzed samples were 0.0%, 1.5%, and 2.5% in relation to the flour as a whole. Gelatinization temperature, peak viscosity, trough, breakdown, final viscosity, and setback were determined (Figure 4).

Our studies confirm the influence of salt addition on changes in parameters during starch gelatinization in wheat flour (Table 6). The pasting temperature of starch in wheat flour with the addition of salt was higher than in samples without salt. It was also found that the higher the salt addition, the higher the viscosity, both maximum and final, of the flour suspension noted.

There are various types of salt intended for direct consumption or for food purposes, but only some of them are marked with European geographical indications.

## 6. Salts with Geographical Indications

With increasing consumer awareness, information about a product’s place of origin has transformed from a minor detail (included in small print on packaging) into one of the main marketing slogans. Connecting a product with a region is meant to encourage consumers to purchase it. Consumers’ motives when purchasing a product from a given location vary. These may include positive associations with the high quality of products originating from a given country or a sense of “consumer patriotism” (the positive impact of the choice on the national community with which the consumer identifies) [118].

According to surveys conducted by Chudy et al. [119], 85% of consumers consciously choose products from a specific region.

**Figure 5:**
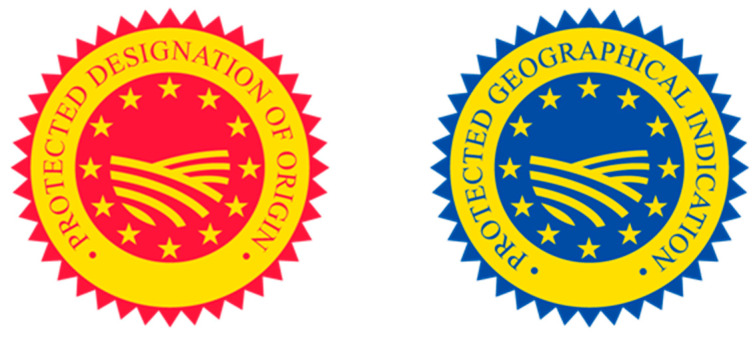
Labels of protected designation of origin (PDO) and protected geographical indication (PGI) [120].

Since 1992, the EU has had a system for registering and protecting regional product names. The basic symbols used to identify agricultural products and foodstuffs are those shown in Figure 5. They are protected designation of origin (PDO) and protected geographical indication (PGI). The PDO label is assigned for products where all stages of production take place in the defined geographical area, and PGI is assigned when at least one stage of production takes place in the defined geographical area. European geographical indications serve as a system for the protection and promotion of products that have a specific origin and possess characteristics associated with that origin. The primary goal of EU geographical indications is to promote products from specific regions. They also aid in maintaining cultural heritage, local customs, and distinct methods of production [119].

Producers who use these marks to mark their products emphasize the product’s connection with a given country, region, or town, which is often the main factor for the buyer when choosing a product. The distinctiveness of products frequently stems from the specific natural circumstances of a particular area or the historical methods of their creation, and this is true for salt as well.

Table 7 presents 11 salts with the PDO mark (including 1 at the application stage; the 1st one in the table) and 7 salts with the PGI mark (including 2 at the application stage; the 13th and 17th in the table) [121].

In Europe, only eight countries (Croatia, France, Greece, Ireland, Italy, Portugal, Turkey, and the United Kingdom) can demonstrate such geographical indications. Germany and Poland, despite being leading salt producers with rich salt traditions, unfortunately, do not have certified salt.

## 7. Conclusions

The review of the scientific literature indicates the following:

1. There is a need to define the parameters of micronized salt, along with performing further toxicological studies on contaminants and additives in salt;
2. There is a need to analyze the long-term health effects, and to develop techniques for removing microplastics in salt and eliminating plastic packaging;
3. In medicine, there are still no clear answers as to how much sodium a healthy body can accumulate;
4. The wider use of fluoride in salt should be considered as an interesting solution for anti-caries prophylaxis;
5. Further research on compounds added to salt to supplement iodine is necessary;
6. In order to show national and cultural heritage and promote regions with a long tradition of salt production, it is advisable for salt producers to apply for the protection of their products with the PGI or PGI mark.

Furthermore, the presented experiment demonstrated that salt affects both the gluten–proteolytic and starch–amylolytic systems in wheat dough. Its addition increases dough stability and extends its development time. Salt also influences the starch gelatinization process, increasing the gelatinization temperature and paste viscosity.

## Figures and Tables

**Table 1:** List of countries with the largest salt production in 2023, by the United States National Minerals Information Center.

Number	Country	Production [Million Tons/Year; 10^9^ kg/Year]		
		2003	2013	2023 ^e^
1	China	32.4	70.0	54.0
2	U.S.	43.7	40.3	42.0
3	India	15.0	16.0	27.0
4	Germany	15.7	11.9	15.0
5	Australia	9.8	11.0	12.0
6	Canada	13.3	12.2	12.0
7	Mexico	8.0	10.8	8.6
8	Chile	nd	6.6	10.0
9	Turkey	nd	5.3	9.1
10	Russia	2.8	nd	8.2
11	Brazil	6.1	7.5	6.6
12	Netherlands	nd	nd	5.3
13	France	7.0	6.1	4.6
14	Poland	1.5	4.4	4.5
15	Spain	3.2	4.4	3.9
16	Ukraine	2.3	6.2	nd
World total		210	262	270

e—estimated data, nd—no data.

**Table 2:** Salt additives.

Chemical Compound	Symbol INS	Maximum Level	Year Adopted
Calcium carbonate	170 (i)	GMP	2006
Calcium silicate	552	GMP *	2006
Sodium ferrocyanide	535	14 mg/kg	2006
Potasum ferrocyanide	536	14 mg/kg	2006
Calcium ferrocyanide	538	14 mg/kg	2006
Magnesium carbonate	504 (i)	GMP	2006
Magnesium oxide	530	GMP	2006
Magnesium silicate, synthetic	553 (i)	GMP	2006
Methacrylate copolymer, basic	1205	GMP	2021
Phosphates	varied	8800 mg	2006
Polysorbates	432–436	10 mg/kg	2006
Salts of myristic, palmitic and stearic acids with sodium, potassium, calcium and ammonia	470 (i)	GMP	2006
Silicon dioxide	551	GMP	2006
Sodium aluminium silicate	554	1000 mg/kg	2013

* Good manufacturing practice.

**Table 3:** Average salt contents in dairy, cereal, seafood, and meat products (%).

Product	Salt Content	Source
Cheese	0.5–2.0	[72]
Mold cheese	3.0–7.0	
Butter	2.16 (2–6% in Brazil)	[73]
Wheat-rey bread	0.90	[74]
Wheat bread	0.60	
Wheat rolls	0.53	
Shellfish and seafood	1.20	[75]
Sausages and charcuterie	2.20	

**Table 5:** Farinographic characteristics of wheat dough without and with salt addition *.

Product Feature	Dough with 0.0% NaCl	Dough with 1.5% NaCl	Dough with 2.5% NaCl
Water absorption [%]	63.6	63.5	62.3
Dough Development Time [min]	2.3	2.3	5.2
Dough Stability [min]	2.9	1.0	18.0
Degree of Softening [BU]	60	65	2
Farinograph Quality Number (FQN) [mm]	38	26	200

* Unpublished results of the authors.

**Table 6:** Pasting properties of wheat dough with the addition of salt (0.0–2.5% related to flour) *.

Product Feature	Dough with 0.0% Salt	Dough with 1.5% Salt	Dough with 2.5% Salt
Pasting temperature [°C]	60.00	61.75	61.85
Peak Viscosity [cP]	2547	2771	2916
Trough [cP]	1577	1645	1684
Breakdown [cP]	970	1126	1232
Final Viscosity [cP]	2880	2893	3065
Setback [cP]	1303	1248	1381

* Unpublished results of the authors.

**Table 7:** Salts with geographical indications (Product type: Food; Class 2.6 Salt).

No	Name and Number of the Registration File	Type of Indication
1	អំបិលកំពតកែប/Kampot-Kep Salt; PGI-KH-03286	PGI
2	Paška sol; PDO-HR-02178	PDO
3	Sel de Salies-de-Béarn; PGI-FR-01311	PGI
4	Sel de Camargue/Fleur de sel de Camargue; PGI-FR-02443	PGI
5	Sel de l’Île de Ré/Fleur de sel de l’Île de Ré; PGI-FR-02782	PGI
6	Sel de Guérande/Fleur de sel de Guérande; PGI-FR-0861	PGI
7	Aφρίνα/Afrina; PGI-GR-02822	PGI
8	Garam Amed Bali/Bunga Garam Amed Bali; PDO-ID-02610	PDO
9	Achill Island Sea Salt; PDO-IE-02652	PDO
10	Oriel Sea Salt; PDO-IE-01318	PDO
11	Oriel Sea Minerals; PDO-IE-01319	PDO
12	Sale Marino di Trapani; PGI-IT-0892	PGI
13	Sal de Castro Marim/Flor de Sal de Castro Marim; PDO-PT-02607	PDO
14	Sal de Tavira/Flor de Sal de Tavira; PDO-PT-0913	PDO
15	Sal de Rio Maior/Flor de Sal de Rio Maior; PDO-PT-02589	PDO
16	Piranska sol; PDO-SI-1098	PDO
17	Delice Doğal Kaynak Tuzu; PDO-TR-03223	PDO
18	Anglesey Sea Salt/Halen Môn; PDO-GB-1068	PDO

## Data Availability

No new data were created or analyzed in this study. Data sharing is not applicable to this article.
